# Role of Vegetation-Associated Protease Activity in Valve Destruction in Human Infective Endocarditis

**DOI:** 10.1371/journal.pone.0045695

**Published:** 2012-09-20

**Authors:** Ghada Al-Salih, Nawwar Al-Attar, Sandrine Delbosc, Liliane Louedec, Elisabeth Corvazier, Stéphane Loyau, Jean-Baptiste Michel, Dominique Pidard, Xavier Duval, Olivier Meilhac

**Affiliations:** 1 INSERM U698, Paris, France; 2 Cardiovascular Surgery Department, Bichat Hospital, AP-HP, Paris, France; 3 University Paris Diderot, Sorbonne Paris Cité, Paris, France; 4 Institut National des Sciences du Vivant, Centre National de la Recherche Scientifique, Paris, France; 5 INSERM CIC 007, Paris, France; 6 Bichat Stroke Centre, AP-HP, Paris, France; National Institutes of Health, United States of America

## Abstract

**Aims:**

Infective endocarditis (IE) is characterized by septic thrombi (vegetations) attached on heart valves, consisting of microbial colonization of the valvular endocardium, that may eventually lead to congestive heart failure or stroke subsequent to systemic embolism. We hypothesized that host defense activation may be directly involved in tissue proteolytic aggression, in addition to pathogenic effects of bacterial colonization.

**Methods and Results:**

IE valve samples collected during surgery (n = 39) were dissected macroscopically by separating vegetations (VG) and the surrounding damaged part of the valve from the adjacent, apparently normal (N) valvular tissue. Corresponding conditioned media were prepared separately by incubation in culture medium. Histological analysis showed an accumulation of platelets and polymorphonuclear neutrophils (PMNs) at the interface between the VG and the underlying tissue. Apoptotic cells (PMNs and valvular cells) were abundantly detected in this area. Plasminogen activators (PA), including urokinase (uPA) and tissue (tPA) types were also associated with the VG. Secreted matrix metalloproteinase (MMP) 9 was also increased in VG, as was leukocyte elastase and myeloperoxidase (MPO). The presence of neutrophil extracellular traps (NETs) associating MPO and externalized nucleosomes, was shown by immunostaining in the VG. Both MPO and cell-free DNA were released in larger amounts by VG than N samples, suggesting bacterial activation of PMNs within the vegetation. Finally, evidence of proteolytic tissue damage was obtained by the release of fragments of extracellular matrix components such as fibrinogen and fibronectin, as well as protease-sensitive receptors such as the uPA receptor.

**Conclusion:**

Our data obtained using human IE valves suggest that septic vegetations represent an important source of proteases originating from massive leukocyte recruitment and activation of the host plasminergic system. The latter forms a potential therapeutic target to minimize valvular tissue degradation independently from that induced by bacterial proteases.

## Introduction

Infective endocarditis (IE) is characterized by vegetative lesions consisting of microbial colonization of a damaged valvular endocardium (native valve IE), or of an intracardiac prosthesis (prosthetic IE). IE is associated with high mortality rates (15–20%), and high costs, related to the duration of antibiotic therapy and the need for surgery in half of the patients [1], [2]. The infected vegetation is the elementary lesion of IE. It is formed by successive appositions of fibrinoplatelet thrombi, incorporating pathogens and leukocytes [3]. The characteristics of the bacterial population that colonizes the vegetation may explain the limited efficacy of antibiotics sometimes observed in the treatment of human IE. After the initial phase of bacterial adhesion and colonization, pathogens proliferate within the forming vegetation. The non-homogenous distribution of bacteria within the vegetation often leads to formation of “clusters” protected by layers of fibrin which constitute an important morphological feature of the lesion [3].

Compared with fibrinoid vegetations of IE, the structure and biology of non-infected thrombi, such as those observed in human abdominal aortic aneurysms (AAA), have been studied in more detail. These mural thrombi which maintain a constant interface with the circulating blood, are a source of proteases [4], including matrix metalloproteinases (MMPs), leukocyte elastase [5], and proteases of the plasminergic system [6]. These host proteases are capable of inducing both degradation of extracellular matrix (matrilysis) and apoptosis of smooth muscle cells subsequent to the loss of cell adherence (anoikis) [5], [6], [7]. For example, laminin, vitronectin, and fibronectin can be cleaved by plasmin and elastase. In IE vegetations, proteolytic and pro-oxidant activities associated with leukocyte activation are similar to those observed in uninfected mural thrombi (i.e. AAA) [4], although the infectious nature of thrombi in IE is responsible for a greater biological activity due to enzymatic activities of the microorganisms themselves, but also due to the massive recruitment of innate immune cells. This recruitment of leukocytes may therefore constitute an additional deleterious insult, related to the cytotoxicity of proteolytic enzymes or radical forms of oxygen [8]. Moreover, bacteria may also represent a source of proteases, including matrilytic gelatinases [9] that can induce vascular cell anoikis [10], [11]. They can also be procoagulant (staphylocoagulase) and fibrinolytic (staphylokinase and streptokinase are able to convert plasminogen into plasmin) [12]. The generation of plasmin by bacterial plasminogen activators may also favor weakening of the surrounding tissue and thus promote bacterial invasion.

The aim of this study was to characterize leukocyte-driven proteolytic activity as well as proteases associated with thrombus formation/degradation, and to assess their pathogenic effect on the valvular tissue/myocardium. We thus analyzed the septic thrombi that constitute the vegetations in human endocarditis by histological and biochemical approaches.

## Methods

### Tissue Samples

Thirty-nine valve samples were obtained from patients operated for IE, conforming with the principles outlined in the Declaration of Helsinki. The study was approved by INSERM Ethics Committee. Before surgery, all patients were informed that surgical wastes would be used for this study. A verbal consent was obtained from each patient and a certificate of non-opposition was signed by the treating physician. This is the standard procedure for the use of surgical waste for research purposes according to the French ethical laws (L. 1211-2 to L. 1211-7, L. 1235-2 and L. 1245.2 - August 6th 2004). **(** **Table 1** **)**.

**Table 1:** Demographic data of the patients.

Characteristic	
Sex (males/females)	n = 30/6
Age (years)	56 (21–82)
Valve (mitral/aortic)	n = 15/24
Causative microorganism	
• Staphylococci	n = 4
• Streptococci	n = 29
• Haemophilus Influenzae	n = 2
• Bartonella	n = 1

Tissue samples were collected in saline and processed within 2 hours after surgery. No antiprotease treatment was used to allow subsequent assessment of tissue proteolytic activities. Patients were from 21 to 82 years old. None of the patients were on anticoagulant treatment. Twenty-one patients had received antibiotics during the 2 weeks preceding the surgery.

### Dissection of Valves into Vegetation and Adjacent Apparently Normal Parts

**Figure 1:**
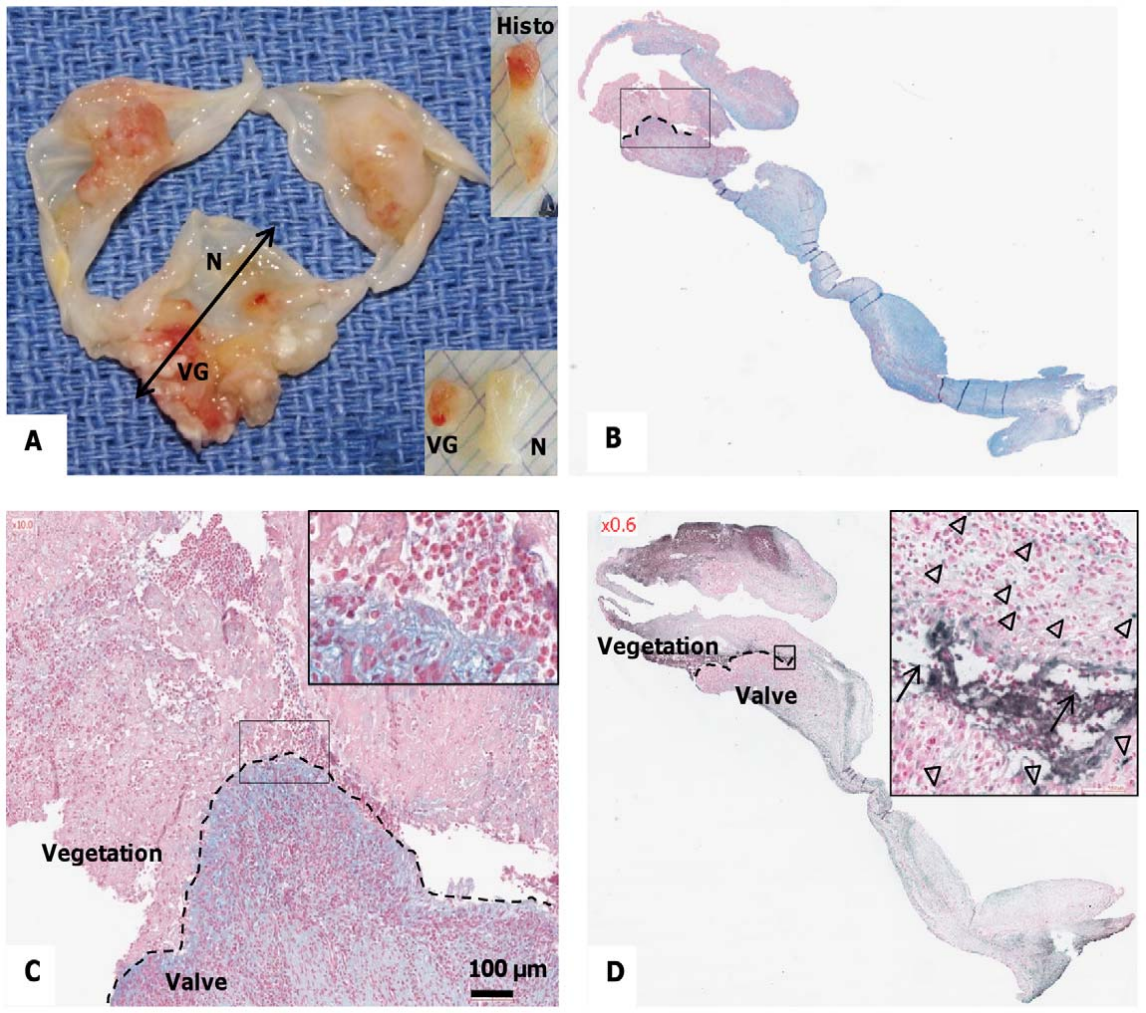
Characterization of the excised valve(s) containing infected vegetations. (A) Macroscopic view of human endocarditic valves, sectioned for histological analysis (“Histo”) as indicated by the double-headed arrow. Vegetations (VG) and adjacent macroscopically normal (N) parts of the valve were then incubated in culture medium for preparation of “conditioned medium”. (B) Alcian blue staining with nuclear fast red counterstaining of a vegetation and underlying valvular tissue. Blue areas correspond to mucoid degeneration interfacing with infected thrombus (rectangle, magnified in C). Red staining shows inflammatory cell nuclei as well as the septic thrombus. (C) Higher magnifications of the interface between the vegetation and the valvular tissue (Alcian blue staining). Inset shows an accumulation of cells with polylobed nuclei. (D) Detection of fragmented DNA by TUNEL showing apoptotic cells (arrowheads) and extracellular fragmented DNA (arrows). Nuclei were counterstained with nuclear fast red.

A representative part of the valve was fixed in 3.7% (w/v) paraformaldehyde for histological analysis, whereas the remaining part was dissected macroscopically by separating the vegetation (VG) itself and the surrounding damaged valvular tissue from the adjacent, apparently normal (N) valvular tissue (**Figure 1**).

### Conditioned Media

The pieces of fresh VG and N tissue samples were weighed and then incubated separately in RPMI (Gibco, Grand Island, NY) containing antibiotics for 24 h at 37°C in a ratio of 10 µL per mg of wet tissue [8]. Conditioned media were then collected and centrifuged (3000 g for 10 min at 20°C). Supernatants were stored at −80°C until further analysis, and the tissue samples were frozen. Tissue conditioned media were then used for determination of secreted protease activity, and for assessment of the presence of soluble fibrinogen, fibronectin and uPAR fragments. Analyses were performed on all samples available for the each experiment, limiting the number of samples in certain cases as detailed in **Figure S1**.

### Immunohistochemistry and Immunohistofluorescence

Paraformaldehyde-fixed endocarditic valve samples were embedded in paraffin, and cut into 5 µm-thick sections. Immunohistochemistry was performed using primary antibodies targeting platelet-specific marker, GPIb (anti-CD42b; dilution 1∶200, Immunotech, Marseille, France), as well as primary antibodies targeting components of the fibrinolytic system: tissue-type plasminogen activator (anti-tPA ESP5, dilution 1∶1000, American Diagnostica, Greenwich, CT), urokinase-type plasminogen activator (anti-uPA #3689, dilution 1∶500, American Diagnostica), uPA receptor (anti-uPAR #3932, dilution 1∶1000, American Diagnostica), and human plasminogen (anti-Pg Ab-1, dilution 1∶20, Neomarker, Fremont, CA). Anti-CD66b (dilution 1∶25, Immunotech, Marseille, France) to detect granulocytes, anti-elastase (dilution 1∶100, HBT, HM2174, Uden, The Netherlands) and anti-MMP9 (dilution 1∶50, Abcam, ab58803, Cambridge, UK) antibodies were also used.

Antigen retrieval was performed with Tris/EDTA, pH 9.0 (anti-CD42b, anti-tPA, anti-Pg, anti-CD66b, anti-MMP-9) or with citrate 0.1 M, pH 6.0 (anti-uPA, anti-uPAR, anti-elastase) at 95°C, endogenous peroxidase was blocked with 3% (v/v) H_2_O_2_ in aqueous solution, and nonspecific binding was blocked with 1% (w/v) bovine serum albumin. Sections were incubated overnight with the primary antibody, followed by a secondary antibody (Kit LSAB, Dako A/S, Glostrup, Denmark). Horseradish peroxidase-conjugated antibodies were detected by 3′-diaminobenzidine tetrahydrochloride. Nuclei were then counterstained with Papanicolaou stain. Control irrelevant antibodies (Dako) were applied at the same concentration to assess nonspecific staining.

For immunofluorescence, sections were incubated for 1 h with the primary antibody, anti-myeloperoxidase (MPO) (rabbit polyclonal antibody, dilution 1∶100, Dako A0398) or anti- eosinophil major basic protein (EMBP) (mouse monoclonal antibody, dilution 1∶50, Santa Cruz Biotechnology, sc-59164) at room temperature followed by rinsing and incubation with the appropriate secondary antibody (respectively goat anti-rabbit IgG or goat anti-mouse IgG) coupled to AlexaFluor ® 555 (Invitrogen, Carlsbad, CA) for 1 h at room temperature. DAPI (4′,6′-diamidino-2-phenylindole, 100 ng/mL, Sigma Aldrich) was used for nuclear staining. Paraffin sections were also stained with Alcian blue (nuclear fast red counterstaining) to evaluate the general topography of the tissue and the cell distribution.

### Detection of Apoptosis *in situ*

Valve sections were subjected to terminal deoxynucleotidyl transferase dUTP nick end labeling (TUNEL) assay. TUNEL assay was performed using TACS®2 Tdt- Blue Label TUNEL Kit (Trevigen, Gatherburg, MD), according to the manufacturer’s protocol. HistoGreen/HISTOPRIME® (AbCys, Paris, France) was used as substrate for HRP. Green positivity of apoptotic nuclei was evaluated under a light microscope.

### Gelatin Zymography

Gelatinolytic activity in conditioned media were measured as previously described [8]. Samples were analyzed by electrophoresis on 10% SDS-PAGE containing 2.5 mg/mL bovine gelatin (Sigma Aldrich, Saint Louis, Missouri). Non-MMP gelatinolytic activity was tested using the above method with the addition of 2.5 mg/mL fucoidan (Sigma Aldrich) to the resolving gel. Fucoidan, a sulfated fucosylated polymer from brown algae that exhibits some heparin/heparan sulfate properties, was found in our laboratory to enhance the sensitivity of SDS-PAGE-gelatin zymography for leukocyte elastase detection. Gelatin and gelatin-fucoidan gels were incubated at 37°C for 19 and 36 h, respectively. Activities were quantified by gel densitometry using Image J v.1.42 software (National Institute of Mental Health, Bethesda, MD). A standard sample (supernatant of activated human neutrophils) was run on each gel and served as reference. Chemicals for SDS-PAGE were obtained from Sigma or from Bio-Rad (Hercules, CA).

### Western Blot Analysis

Standard procedures were used for Western blot analysis. Soluble fragments of fibronectin, fibrinogen, or uPAR, as well as plasmin-antiplasmin complexes were detected in conditioned media after protein separation by SDS-PAGE followed by transfer to polyvinylidene fluoride membranes (PVDF, Hybond-P, Amersham/GE Healthcare, Little Chalfont, England). The membranes were then incubated with antibodies to human fibronectin (rabbit polyclonal F3648, dilution 1∶12000, Sigma Aldrich), human fibrinogen (rabbit polyclonal A0080, dilution 1∶5000, Dako), uPAR (mouse monoclonal #3932, dilution 1∶2500, American Diagnostica), and human plasminogen (Anti-Pg Ab-1, dilution 1∶200, Neomarker) for 16 hours at 4°C. Appropriate peroxidase-conjugated secondary antibodies (AffiniPure, Jackson Immunoresearch, West Grove, PA) and chemiluminescence detection kits (ECL, Amersham/GE Healthcare) were then used for antigen-antibody complexes detection, and membranes were finally exposed to X-ray films (Hyperfilm, Amersham/GE Healthcare). A standard human protein sample was run on each gel and served as reference.

**Figure 2:**
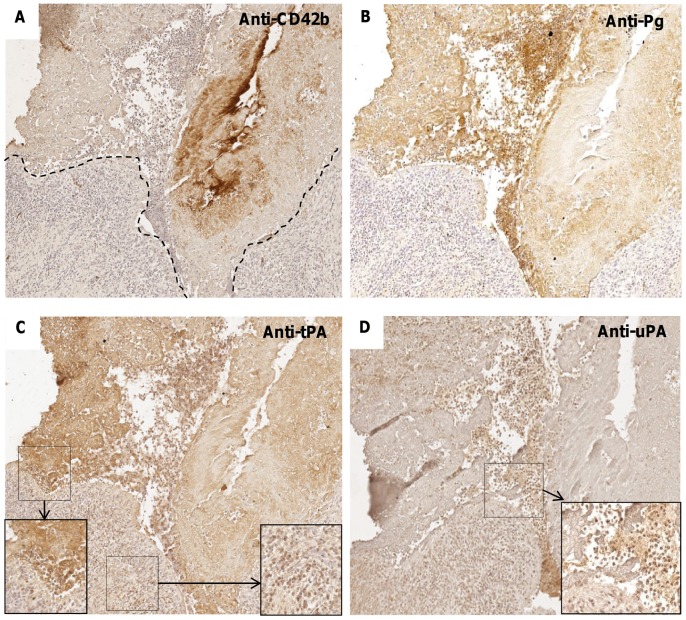
Immunostaining for platelet markers and PAs in human endocarditic valves. (A,B) Immunostaining for CD42b/GPIb and for plasminogen, respectively, with hemalun nuclear counterstaining (x10), showing positive immunostaining in the VG. (C) Immunostaining for tissue-type PA (tPA). Insets: higher magnification (x20) of the interface VG/valve (left-hand side) and within the valvular tissue (right-hand side). (D) Immunostaining for urokinase (uPA). Inset: higher magnification (x20) of the interface VG/valve showing an intense staining of polymorphonuclear cells.

### Plasmin Activity

Conditioned medium (20 µL) was incubated in 0.05 M HEPES buffer, pH 7.4, 0.75 M NaCl, 0.05% NP40 with 40 µM of the synthetic plasmin substrate, MeOSuc-Ala-Phe-Lys-AMC (Bachem, Torrance, CA). Substrate hydrolysis was monitored for 2 h using a spectrofluorometer (Hitachi F-2000, Braun Science Tec, Les Ulis, France) with excitation and emission wavelengths of 390 nm and 460 nm, respectively.

**Figure 3:**
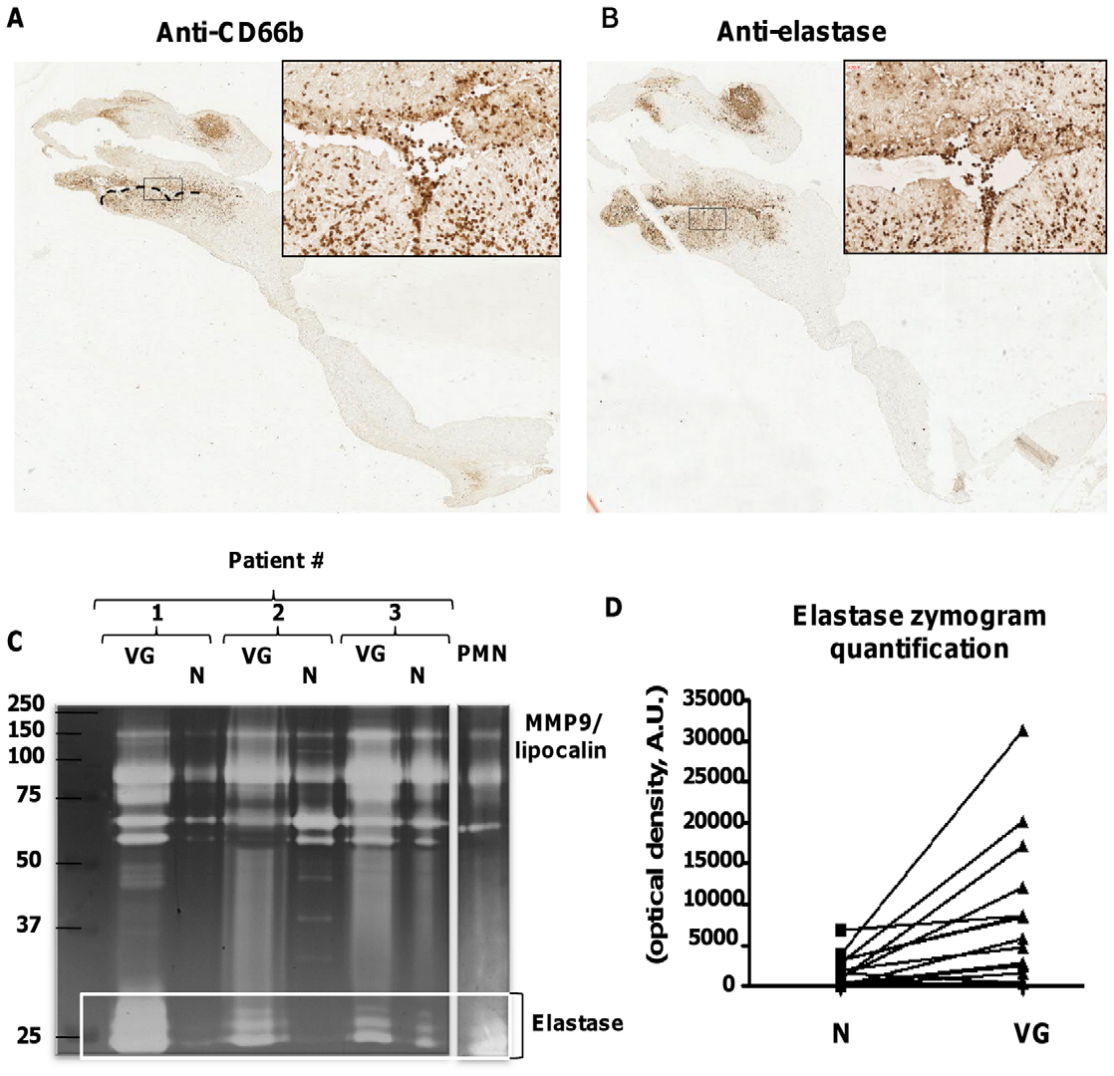
Immunocolocalization of PMNs and leukocyte elastase in human endocarditic valves. (A,B) Immunostaining for CD66b and for elastase, respectively (x1.2), highlighting the presence of numerous PMNs at the interface VG/valve. Inset: higher magnification of these areas (x20). (C) Representative zymogram of conditioned media of VG and N from three patients. Supernatant of activated PMNs (20 µL) was used as a control for secreted elastase. Positions in the gel of molecular mass standard proteins used for calibration are indicated on the left-hand side. (D) Quantification of zymograms: areas of gelatinolysis corresponding to elastase were quantified by densitometry for 17 pairs of conditioned media. The mean optical intensity is 1572±460 *versus* 7549±2075 for N and VG samples, respectively, (*P* = 0.0001).

### ELISA

Leukocyte elastase/α_1_-antitrypsin (AAT) complexes and human MPO concentrations were determined in duplicate with commercially available ELISA kits (Calbiochem (Darmstadt, Germany) and Hycult Biotechnology (CliniSciences S.A.S, Nanterre, France), respectively.

### Cell-free DNA Assay

Quantification of cell-free DNA (cf-DNA) was carried out in duplicate using Quant-it™ Picogreen® ds DNA Reagent (Invitrogen) as previously described [13].

### Determination of Bacterial Endotoxin

Endotoxins in the conditioned media (n = 11) were quantified using the Limulus Amebocyte Lysate (LAL) chromogenic endpoint assay (Hycult Biotechnology) according to the supplier’s instructions. Briefly, samples diluted at 1∶5 (50 mL final) were incubated with LAL reagent (50 mL) for 30 minutes at room temperature and a stop solution was added before reading on a spectrophotometer at 405 nm.

### Statistical Analysis

Results were analyzed by KaleidaGraph software (KaleidaGraph 4.0, Synergy Software, Reading, USA) and expressed as means ± SEM. Analysis was performed by paired non-parametric (Wilcoxon) test for comparison of VG *versus* N samples. A value of *P*<0.05 was considered statistically significant.

**Figure 4:**
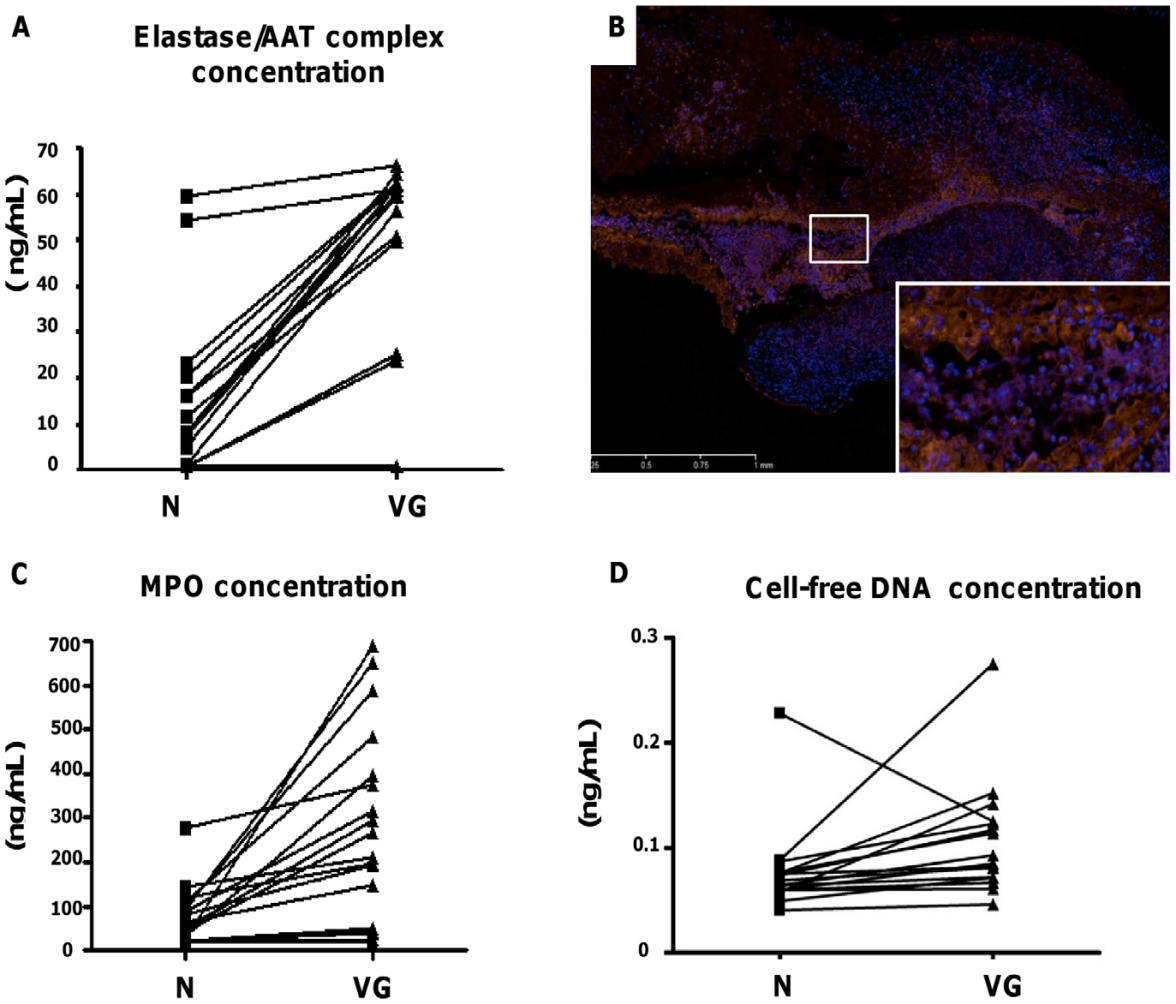
Characterization of protease activity in the vegetation. (A) Diagram illustrating the variation of concentrations of elastase/α_1_-antitrypsin complexes between N and VG for 16 pairs of conditioned media. The mean of concentrations is 0.0769±0.0106 *versus* 0.107±0.014 ng/mL for N and VG respectively, (*P* = 0.004). (B) Immunostaining of MPO (red fluorescence) coupled to DNA detection by DAPI (blue fluorescence) (x20) showing both cellular and extracellular staining at the interface VG/valve. Co-localization of MPO and DAPI staining was observed in extracellular filamentous DNA NETs. (C) Diagram illustrating the variation in extracellular MPO concentrations in the conditioned media between N and VG. The mean of concentrations is 12±4 *versus* 39±6 ng/mL for N and VG respectively, (*P*<0.001). (D) Concentrations of cf-DNA in the conditioned media of N and VG samples. The mean of concentrations is 73±14 *versus* 252±49 ng/mL for N and VG respectively (*P*<0.001).

## Results

### Vegetations and Subjacent Tissue Damage

For each valve collected, one representative part was chosen for histological characterization, the remaining tissue was macroscopically separated into the damaged part comprising the vegetations (“VG”) and the adjacent apparently normal tissue (“N”) for preparation of the conditioned medium **(** **Figure 1A** **)**. Alcian blue staining demonstrated the presence of mucoid degeneration in the valvular tissue underlying the invasive septic thrombus **(** **Figure 1B–C** **)**. Within the vegetation itself, leukocytes, and in particular PMNs, with polylobed nuclei were identified following nuclear fast red staining **(** **Figure 1C** **, inset)**. Finally, TUNEL-positive apoptotic cells were observed at the interface between the vegetation and the underlying tissue **(** **Figure 1D** **, inset, arrowheads)**. In the same area, extracellular fragmented DNA was also positive for TUNEL reaction **(** **Figure 1D** **, inset, arrows)**.

**Figure 5:**
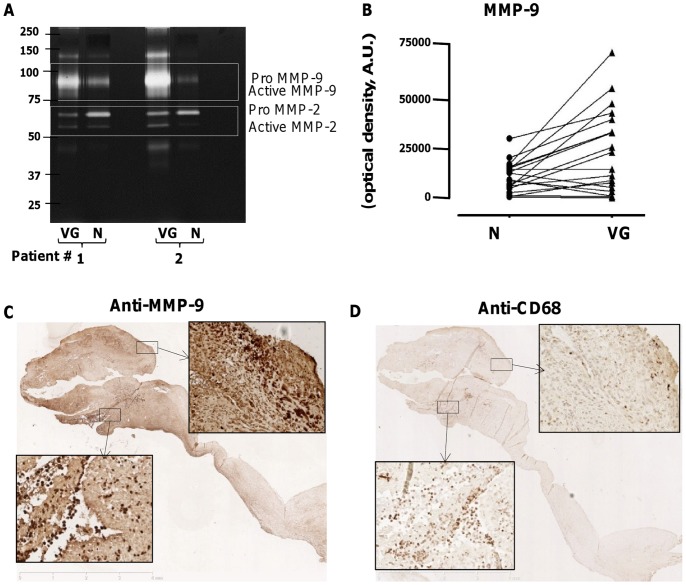
Identification and activity of MMPs and plasmin in human endocarditic valves. (A) Detection of the different forms of MMP-9 and MMP-2 in conditioned media of VG and N, in representative zymogram from 2 patients. Gelatinolytic bands corresponding to proMMP-9, MMP-9, proMMP-2 and MMP-2 were quantified and expressed in arbitrary optical density units. (B) Diagram illustrating the variation of MMP-9 activity between N and VG for 19 pairs of conditioned media (*P*<0.01). (C,D) Immunostaining of MMP-9 and macrophages respectively (x1.2) in human endocarditic valves. (C) Strong immunostaining of MMP-9 in the VG and adjacent valve tissue. Insets: higher magnification (x20) of the interface VG/valve (bottom left) and within the valvular tissue (top right). (D) Presence of numerous macrophages at the interface VG/valve where only few of them could be observed in the upper area. Insets: higher magnification (x20) of the interface VG/valve (bottom left) and within the valvular tissue (top right).

**Figure 6:**
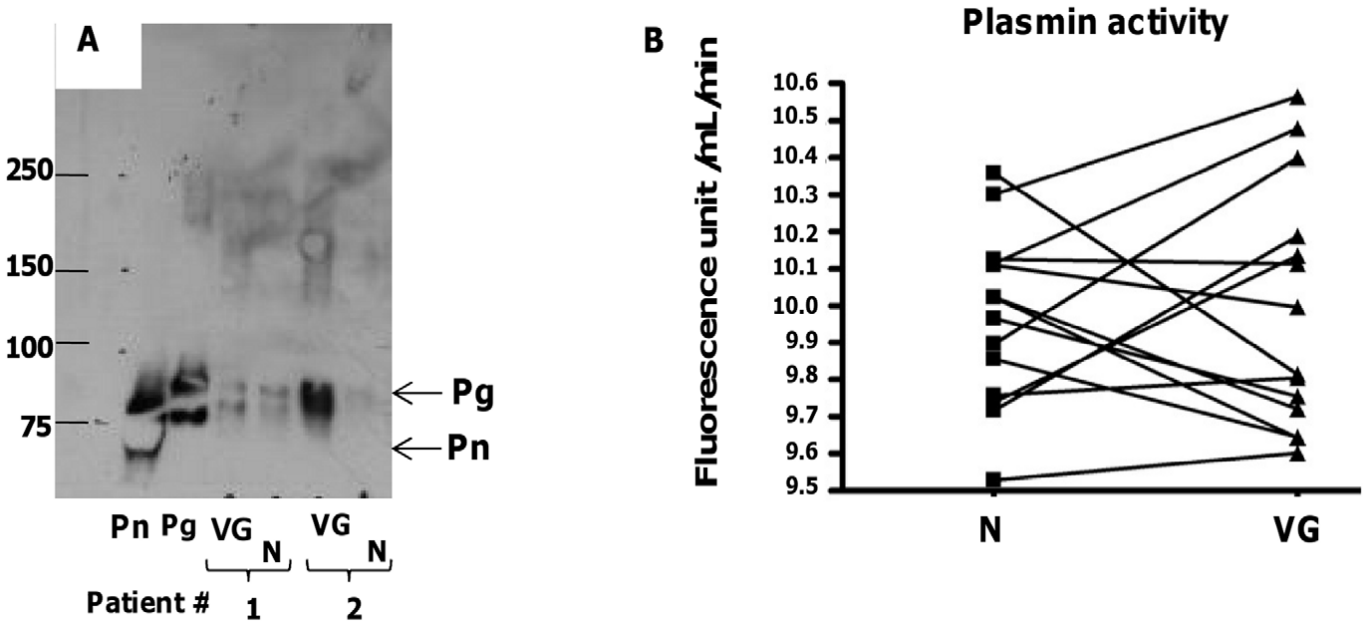
Plasmin activity in the conditioned media. (A) Western blot analysis for detection of plasmin(ogen) with a monoclonal anti-human plasminogen antibody, in conditioned medium obtained from the VG and N samples of 2 representative patients (Pn and Pg: purified human plasmin, 0.25 µg *per* well, and purified human plasminogen 0.2 µg *per* well, respectively, used as reference). Positions in the gel of molecular mass standard proteins used for calibration are indicated on the left. (B) Plasmin activity quantified in the conditioned media of N and VG, using a selective chromogenic substrate (*P* = 0.8).

**Figure 7:**
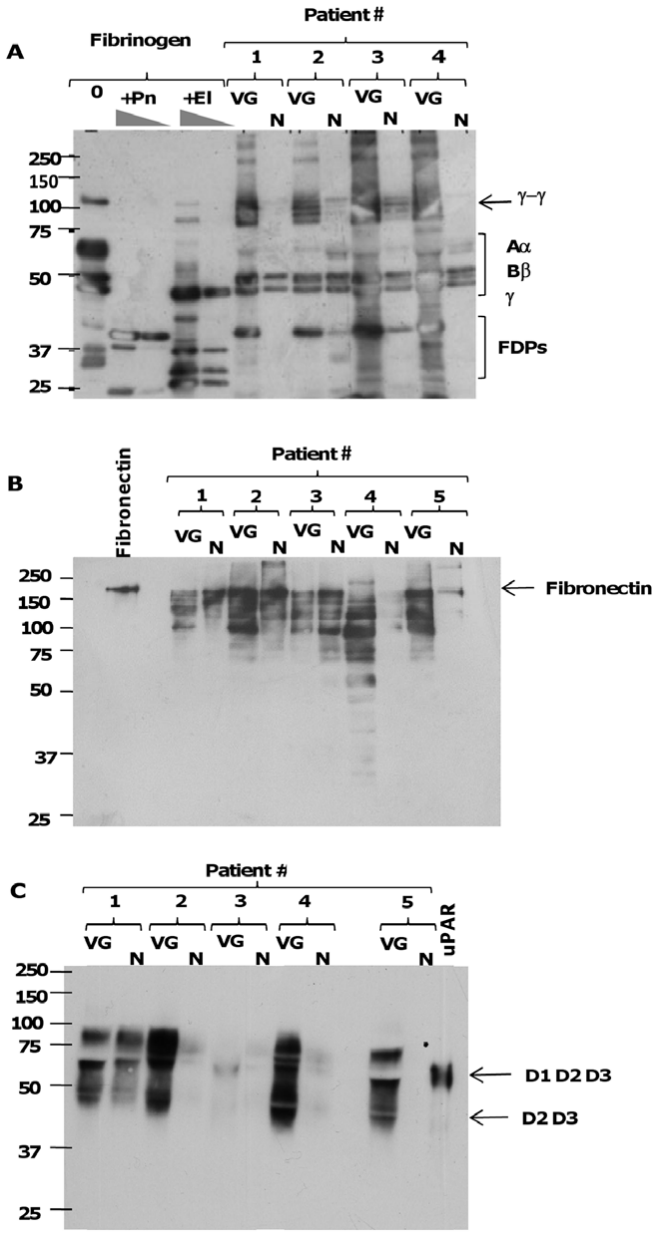
Extracellular matrix and pericellular proteolysis. (A) Western blot analysis of fibrinogen and proteolytic fragments in conditioned media of VG and N. Human fibrinogen (100 µg/mL) was incubated with saline (0), plasmin (Pn, 500 nM and 100 nM) and elastase (El, 100 nM and 20 nM) for 2 h at 37°C, to generate reference fibrinogen degradation products (FDPs). Pairs of conditioned media from 4 patients were analyzed under conditions of reduced disulfide bonds using a rabbit anti-human fibrinogen antibody, which recognizes both the intact Aα, Bβ, and γ chains, γ-γ dimers, and FDPs (positions indicated by brackets on the right-hand side). (B) Western blot analysis of fibronectin and its proteolytic fragments in pairs of conditioned media from 5 patients using a polyclonal anti-fibronectin antibody. Purified fibronectin (0.5 µg *per* well) was used as a control. (C) Western blot analysis of shedding of soluble species of uPAR in pairs of conditioned media from 5 patients, using a mouse monoclonal anti-uPAR D2 domain antibody. Purified recombinant human uPAR (15 ng *per* well) was used as a reference, and the position of the intact three-domain (D1D2D3) and truncated two-domain (D2D3) species under reducing conditions is indicated on the right. For all Western blots, positions in the gel of molecular mass standard proteins used for calibration are indicated on the left.

### Immunohistological Characterization of Platelets and Fibrinolytic System

Immunostaining for the platelet-specific receptor marker CD42b (GPIb) was positive in the vegetation of 35/39 valves **(** **Figure 2A** **)**. Positive immunostaining for plasminogen and tPA was predominantly observed in the vegetation of respectively 31 and 38/39 samples analyzed, associated with the fibrin network **(** **Figure 2 B–C** **)**. Immunodetection of the other plasminogen activator, uPA, showed positivity in 23/39 valves (59%), mainly associated with polylobed cells within the vegetation and adjacent valvular tissue **(** **Figure 2D** **)**. Immunohistochemical analysis showed that 80% and 79% of the valves were positive for plasminogen and uPAR, respectively (not shown).

### Protease Activities Associated with PMNs in the Vegetation

Immunohistological staining for CD66b showed presence of infiltrated granulocytes (chiefly PMNs since very few eosinophils could be detected by immunostaining using anti-EMBP antibody, **Figure S2**) within the vegetation but also at the interface between the septic thrombus and the underlying valvular tissue (**Figure 3A**). A strong staining for elastase was shown in the same area (**Figure 3B**) suggesting that this proteolytic enzyme could produce deleterious effects in the case of cellular activation. To test the hypothesis that PMNs may be activated and release proteases, elastase and gelatinase activities were measured in the conditioned media obtained from vegetations (VG) and the adjacent macroscopically normal (N) part of the valve (**Figure 3C–D**). Gelatin zymography, performed in 17 pairs of conditioned media, showed that VG released significantly more elastase than corresponding normal adjacent tissues (**Figure 3C**). There were more intense areas of gelatinolysis by elastase in VG than in N samples (**Figure 3D**): 7549±2075 *versus* 1572±460 for VG and N samples, respectively (in arbitrary units, n = 17, *P* = 0.0001).

Elastase/α_1_-antitrypsin (AAT) complexes were measured by ELISA in the conditioned medium. Their concentrations were significantly higher in VG *versus* N samples (*P* = 0.004, **Figure 4A**). Immunofluorescence was used for detection of MPO, another PMN-specific enzyme (**Figure 4B**). Strong staining was observed at the interface between the VG and the underlying tissue, in part associated with extracellular DNA (known as “neutrophil extracellular traps” (NETs), inset **Figure 4B**). Quantification of extracellular MPO by ELISA showed that VG released significantly more MPO than the corresponding normal parts of the valve (12±4 *versus* 39±6 ng/mL for N and VG, respectively, *P*<0.001) (**Figure 4C**). Accordingly, VG released more cf-DNA than N parts of the valves, as assessed by fluorescence DNA labeling in the conditioned media (252±49 *versus* 73±14 ng/mL for VG and N, respectively, *P*<0.001) (**Figure 4D**). The majority of causative microorganisms were streptococci (81%) followed by staphylococci (11%). Accordingly we demonstrated that more MPO was released into conditioned media of valves infected with Streptococci (28.5±5 ng/mL) than with Staphylococci (0.76±0.06 ng/mL) (*P* = 0.006). Concurrently, there was a significant increase in the concentration of extracellular MPO in conditioned media of VG (42±7 ng/mL) compared to N (15±5 ng/mL) when the valve was infected with Streptococci (*P* = 0.01), whereas no difference was found when the valve was infected with Staphylococci (*P* = 0.4). Similarly, more cf-DNA was detected in the conditioned media of valves infected with streptococci (173±34 ng/mL) than with staphylococci (33±5 ng/mL) (*P* = 0.03), and a significant increase in the amount of cf-DNA in the conditioned media between VG (269±55 ng/mL) and N (76±18 ng/mL) was observed when the valve was infected with streptococci (*P*<0.01), while no difference between VG and N was found when the valve was infected with staphylococci (*P* = 0.1).

In addition to elastase, gelatin zymography allowed detection of MMP-9 and MMP-2 in conditioned media of endocarditic valves, showing a significantly increased MMP-9 in VG relative to the normal parts of the valves (10366±1714 *versus* 23716±4726 ng/mL for N and VG, respectively, *P*<0.01) (**Figure 5A–B**). In contrast, MMP-2 activity was not statistically different between these two valve regions (*P* = 0.95). Consequently, the MMP-9/MMP-2 ratio was 2.3-fold higher in the conditioned medium of VG than in that of N samples (*P* = 0.004). These results show that there is no difference in MMP-2 release, this MMP being synthesized by most cell types, including mesenchymal cells, whereas MMP-9, associated with PMN activation, and present in gelatinase granules, was released in large amounts by the VG. Gelatin zymography showed an MMP-9/Lipocalin band in the valve samples as well as in supernatant of activated PMNs (**Figure 3C** **,** **5A**). This 125-kDa complex of MMP-9 and neutrophil gelatinase-associated lipocalin (NGAL) protects MMP-9 from autodegradation [14]. In neutrophils, NGAL and MMP-9 are stored in specific granules, while MMP-9 is also present independently in gelatinase granules [15]. Simultaneously, macrophages were detected by immunostaining at the interface between the valve and the VG (**Figure 5C,D** **)**. However, some MMP-9-positive areas were almost devoid of macrophages and presented cells with polylobed nuclei. Whereas macrophages can express MMP-9, it is likely that PMNs represent the major source of MMP-9, due to their abundance in our samples.

### Soluble Plasmin Activity

Since active MMPs were detected by zymography and plasminogen activators (PAs) were present within the vegetations (see **Figure 2**), we hypothesized that plasmin could be generated within the tissue and thus be detected in the conditioned media. However, only plasminogen but not plasmin could be detected by Western blot (**Figure 6A**). Plasmin activity was measured using a synthetic substrate, in 17 pairs of conditioned media from VG and N samples, and no statistically significant difference in plasmin activity could be found (*P* = 0.8) (**Figure 6B**). However, it should be noted that in 10 of 17 valves studied (60%), plasmin activity was greater in the VG *versus* N regions. The variability of plasmin activity detected in the conditioned media of VG may be due to the presence of inhibitors (such as α_2_-antiplasmin) or due to the trapping of plasmin by fibrin or cell membranes within the tissue.

### Tissue Proteolysis in the Infected Region of the Valve

Western blot analysis was performed on VG and N conditioned media in order to evaluate the presence of protein fragments released by protease activities. Using a polyclonal antibody directed against fibrinogen, both γ-γ dimers, resulting from the formation of fibrin, and proteolytic fragments derived from fibrinogen chains, and produced mainly by plasmin and to a lesser extent by elastase, were much more abundant in VG relative to the adjacent undamaged tissue (N), the latter rarely containing traces of intact fibrinogen (n = 12) (**Figure 7A**). Similarly, degradation products of fibronectin were mainly detected in conditioned media obtained from VG, (**Figure 7B**). Densitometric analysis of fibronectin fragments showed that proteolysis was more intense in VG *versus* N samples (data in arbitrary densitometric units, *P*<0.03).

Finally, and as for matrix proteins, the cleavage of membrane uPAR on vascular or blood cells, and the release of its soluble forms (“shedding”) into the conditioned media of the valves were assessed by Western blot using an antibody against the D2 domain of the receptor. Soluble forms of uPAR similar to both its intact three-domain (D1D2D3, m ≈ 60 kDa) and its truncated (D2D3, m ≈ 40 kDa) forms were found to be more abundant in VG than in N samples (**Figure 7C**).

### Presence of Bacterial Endotoxins in the Conditioned Media

Bacterial endotoxins (lipopolysaccharide or LPS) were measured in the media conditioned by VG and N tissue using the Limulus Amebocyte Lysate assay. Out of 11 valves, only 10 of them had detectable levels of bacterial LPS. There was no significant difference between the VG and N conditioned media (2.4±0.98 *versus* 1.3±0.40 for VG and N samples, respectively (in EU/ml), *P* = 0.8. Furthermore, no correlation could be observed between LPS levels and neutrophil activation markers (not shown). One limitation to this approach is that LPS was measured in the conditioned medium and may not directly reflect the bacterial contamination of the valve since part of LPS could remain bound to the tissue (or phagocytosed by neutrophils).

## Discussion

The mechanisms involved in myocardial and valvular injury that can be induced by IE vegetations have not yet been studied in detail. Endothelial injury is probably the initiating factor for platelet deposition and subsequent pathogen colonization upon bacteremia [16]. Intact endothelium is resistant to transient bacteremia and only particular pathogens can directly invade the endothelial layer [17]. In contrast to systemic massive bacteremia such as that observed in sepsis, IE is characterized by focal lesions on the valves initiated either by mechanical excoriation of the endothelium or local inflammatory aggression [3]. This leads to direct contact between blood and bacteria on the one hand, and the subendothelial components, including extracellular matrix proteins and factors of coagulation, on the other hand [3]. The thrombus that forms on the damaged endothelium contains large amounts of plasma proteins such as fibrinogen, fibronectin, vitronectin, and blood cells including red blood cells, platelets and leukocytes that participate in both coagulation and pathogen fixation. The pathogens typically associated with IE indeed bind avidly to these structures rich in platelets, fibrin and various adhesive proteins, and can colonize them during transient bacteremia [18]. We report the presence of platelets, reflecting the continuous renewal of the infected thrombus at the interface between the VG and the blood flow. Platelets favor the recruitment of leukocytes expressing tissue factor, *via* interaction of platelet P-selectin with leukocyte PSGL-1, but also allow direct adhesion of certain bacteria *via* platelet-associated fibrin(ogen) and integrin α_IIb_β_3_ [19]. In this context, experimental models have demonstrated the efficacy of anti-platelet agents in reducing the size of vegetations and their bacterial titer [20]. Retrospective studies have shown a positive impact of antiplatelet agents on embolization [21] and mortality in patients with IE [22]. However, a randomized clinical trial did not show any benefit of aspirin treatment [23]. Integrin α_IIb_β_3_ appears to be an interesting target, because it is involved in adhesion of bacteria as well as leukocytes [24]. We also showed the accumulation of PMNs within the VG, but also their infiltration in the valvular tissue. PMNs have been recently reported to play an important role in thrombus formation *via* serine proteases associated with NETs [25]. Previous studies have shown that septic vegetations displayed higher procoagulant activity than sterile vegetations in an experimental model of IE [26]. Neutrophil elastase and cathepsin G associated with extracellular nucleosomes promote coagulation by proteolysis of the tissue factor pathway inhibitor [25]. In addition to these procoagulant activities, PMNs express the plasminogen activator uPA that may, *via* plasmin formation, induce fibrinolysis and tissue proteolysis. Plasmin, in addition to its fibrinolytic role, is indeed able to degrade extracellular matrix proteins such as fibronectin [27], leading to cell death by anoikis [6]. It has also the ability to convert pro-MMPs into active MMPs [28]. MMPs, in turn, have proteolytic activity on fibrillar networks and are thus able to degrade the extracellular matrix. They may also induce apoptosis as already described for murine endocardial endothelial cells [29]. However, no evidence of induction of apoptosis of myocytes by MMPs, including MMP-2 and MMP-9, has been provided. In an experimental model of IE in rats, we recently demonstrated that purified plasmin and elastase can induce apoptosis of myocytes *ex* *vivo,* independently of bacterial proteases (unpublished data). In this model, the vegetations were able to induce detachment of adherent cells *in vitro*, suggesting that proteases contained in the vegetation could induce extracellular matrix degradation and subsequent cell detachment and death. Most of studies on IE point to bacterial proteases, including gelatinases and activators of plasminogen [30], [31]. However, we have previously reported that non-infected thrombi may represent an important source of protease activity, potentially deleterious for the vascular wall and impeding cicatrization [4], [5], [32]. We recently published that in human abdominal aortic aneurysms (AAAs) bacterial material could be detected in most of thrombus and arterial wall samples [13]. The presence of periodontal pathogens (assessed indirectly by quantification of antibodies to P. gingivalis) correlated with the thrombus volume. In an animal model of AAA, injection of P. gingivalis was shown to induce greater aortic dilatation, associated with important neutrophil recruitment. This was independent of the bacterial proteolytic arsenal since LPS produced a similar effect. In IE, in addition to bacterial protease activities, the massive recruitment and activation of PMNs may therefore play an important role in tissue damage associated with septic vegetations. PMN proteases such as elastase and cathepsin G could induce valvular cell death by pericellular proteolysis, as described for other vascular cells with plasmin and elastase [6], [7], [33]. Here, we report that septic vegetations release more elastase, MMP-9 and MPO than the corresponding adjacent apparently normal area of the valve. This confirms that PMNs have been recruited and activated within the vegetation. The presence of extracellular DNA associated with MPO, as shown in this study by immunofluorescence, reflects an over-activation, beyond degranulation, of PMNs leading to the formation of NETs, potentially procoagulant and displaying protease activity. Whereas NETs are thought to possess bactericidal functions [34], it is not excluded that they can induce damage to the surrounding tissue. Quantitatively, more MPO and cf-DNA were released in the conditioned medium when IE was caused by streptococci than by staphylococci. *S. aureus* has been shown to secrete a nuclease able to digest NETs that could explain the moderate release of cf-DNA and associated MPO during the incubation [35]. This result should be verified with a larger number of samples. In our study, only 4 valves were shown to be infected by staphylococci.

MPO released by PMNs is able to activate plasmin formation by oxidative inhibition of plasminogen activator inhibitor type 1 (PAI-1) and to generate cytotoxic aldehydes from glycine (formaldehyde) and threonine (acroleine) [36]. A combination of proteolytic and oxidative insults resulting from the host response to infection may therefore participate in valvular tissue degradation.

A proof of the activity of these proteases *in situ* in the valve tissue is provided by Western blot analysis, which demonstrated the presence of degradation products of fibrinogen and fibronectin in the conditioned media, released in larger amounts by the vegetation relative to the adjacent area. The degradation of matrix proteins is accompanied by significant pericellular proteolysis, which may participate in apoptotic cell death and cardiac and vascular decellularization inducing functional impairment of the tissue in contact with the vegetation. uPAR is a pleiotropic receptor that is central to cell migration and tissue remodeling, especially during infections and inflammation. In addition to binding to uPA, uPAR also participates in cell adhesion and migration *via* its ability to bind to matrix vitronectin. uPAR functions are regulated by proteases, including plasmin, various MMPs, elastase and bacterial proteases, which cleave and release (shedding) soluble forms of the receptor (suPAR). It has been recently demonstrated that the binding of uPA to its receptor protects endothelial cells from apoptotic death [37]. In our study, immunohistochemistry showed that uPAR was highly expressed at the cellular level in vegetations and adjacent valvular tissue (not shown). Increased soluble forms of uPAR (D1D2D3 and D2D3) in the conditioned media of VG relative to the macroscopically healthy zone support the hypothesis of the presence of active protease activities *in situ* and suggest that cell death may be partly determined by the cleavage/shedding of uPAR.

In conclusion, septic vegetations represent a considerable source of proteases and oxidative injury originating from massive leukocyte recruitment and activation of the plasminergic system. The host response may therefore participate in valvular tissue degradation and enhance bacterial endogenous pathogenic effects.

## Acknowledgments

We thank Dr. Mary Osborne-Pellegrin for help in editing the manuscript. We also thank Dr. Martine Jandrot-Perrus and Dr. Pascal Augustin for their helpful discussion.

## Supporting Information

Figure S1 **Flowchart of human heart valves illustrating type of experimental methods performed on the conditioned media and their distribution.**
(TIF)

Figure S2 **Immunostaining of eosinophils with anti-EMBP antibody with very few cells expressing positive staining.**
(TIF)
