# Supplementary figures and images for: Role of Vegetation-Associated Protease Activity in Valve Destruction in Human Infective Endocarditis
Source: PLoS One. 2012 Sep 20;7(9):e45695. doi: 10.1371/journal.pone.0045695 (PMC3447824; doi:10.1371/journal.pone.0045695)

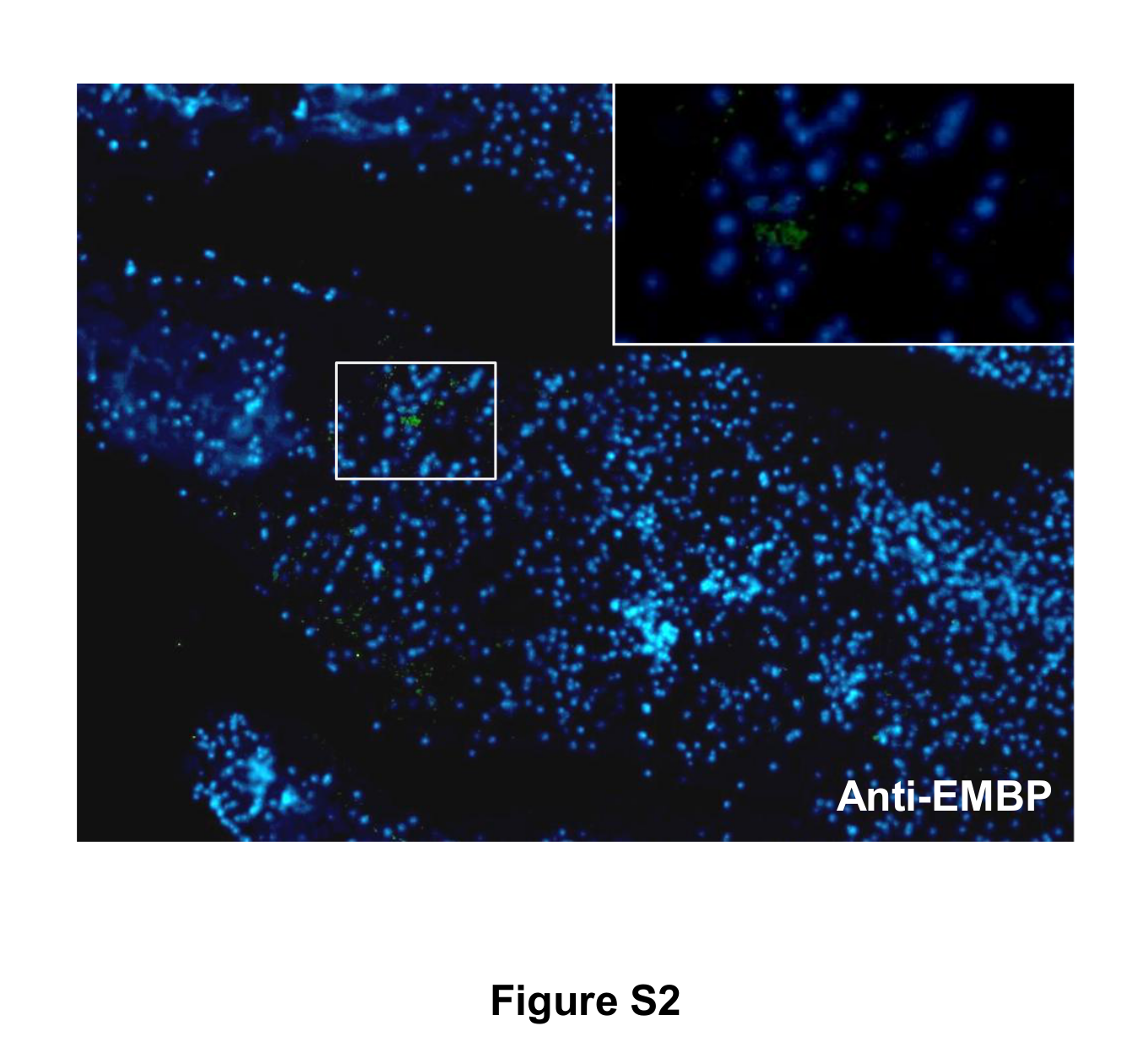

Supplement: Figure S2 — Immunostaining of eosinophils with anti-EMBP antibody with very few cells expressing positive staining. (TIF) [file pone.0045695.s002.tif]
